# Financial considerations in the conduct of multi-centre randomised controlled trials: evidence from a qualitative study

**DOI:** 10.1186/1745-6215-7-34

**Published:** 2006-12-21

**Authors:** Claire Snowdon, Diana R Elbourne, Jo Garcia, Marion K Campbell, Vikki A Entwistle, David Francis, Adrian M Grant, Rosemary C Knight, Alison M McDonald, Ian Roberts

**Affiliations:** 1 Medical Statistics Unit, London School of Hygiene and Tropical Medicine, Keppel Street, London, UK; 2 Centre for Family Research, Free School Lane, Cambridge, CB2 3 RF, UK; 3 Health Services Research Unit, University of Aberdeen, Health Sciences Building, Foresterhill, Aberdeen, UK; 4 Centre for Research and Innovation Management, Brighton, UK; 5 Nutrition and Public Health Interventions Research Unit, London School of Hygiene and Tropical Medicine, Keppel Street, London, UK; 6 Social Dimensions of Health Institute, Universities of Dundee and St Andrews, Airlie Place, Dundee DD1 4HJ, UK

## Abstract

**Background:**

Securing and managing finances for multicentre randomised controlled trials is a highly complex activity which is rarely considered in the research literature. This paper describes the process of financial negotiation and the impact of financial considerations in four UK multicentre trials. These trials had met, or were on schedule to meet, recruitment targets agreed with their public-sector funders. The trials were considered within a larger study examining factors which might be associated with trial recruitment (STEPS).

**Methods:**

In-depth semi-structured telephone interviews were conducted in 2003–04 with 45 individuals with various responsibilities to one of the four trials. Interviewees were recruited through purposive and then snowball sampling. Interview transcripts were analysed with the assistance of the qualitative package Atlas-ti.

**Results:**

The data suggest that the UK system of dividing funds into research, treatment and NHS support costs brought the trial teams into complicated negotiations with multiple funders. The divisions were somewhat malleable and the funding system was used differently in each trial. The fact that all funders had the potential to influence and shape the trials considered here was an important issue as the perspectives of applicants and funders could diverge. The extent and range of industry involvement in non-industry-led trials was striking. Three broad periods of financial work (foundation, maintenance, and resourcing completion) were identified. From development to completion of a trial, the trialists had to be resourceful and flexible, adapting to changing internal and external circumstances. In each period, trialists and collaborators could face changing costs and challenges. Each trial extended the recruitment period; three required funding extensions from MRC or HTA.

**Conclusion:**

This study highlights complex financial aspects of planning and conducting trials, especially where multiple funders are involved. Recognition of the importance of financial stability and of the need for appropriate training in this area should be paralleled by further similar research with a broader range of trials, aimed at understanding and facilitating the conduct of clinical research.

## Background

The system for funding clinical trials in the UK is highly complex and has, in recent years, been subject to a number of changes (Table 1 describes the UK funding model as of December 2005). Trial teams must engage with this evolving system if they are to resource their research. The major sources of funds available in the UK are the public sector funding bodies such as the Medical Research Council (MRC) and the Department of Health's (DH) Health Technology Assessment (HTA) Programme, charitable bodies and industry. In recent decades, concern has been expressed that the funds which are available from the public sector funding bodies, have become severely constrained [3-5]. Increasingly, formal links or partnerships with industry are being built into UK funding strategies to maximise additional support to encourage and facilitate clinical research [6].

**Table 1:** The UK funding system as of December 2006

**Types of costs**	**Definition **(for detailed explanations see [1]).
*Research costs*	These are costs needed in order to run and manage a trial and are met by bodies such as MRC and HTA. They fund the direct costs of the co-ordinating and possibly the recruiting staff (if salaries are not already covered from other sources), equipment, and consumables, as well as indirect costs to cover institutional support.
*NHS support costs*	These costs are associated with delivery and assessment of interventions which occur only during the course of a trial. These include the means to deliver trial interventions to participants, the costs of hospital stays and of additional clinician time. They also include clinical assessments such as scans, X-rays and physical examinations.
*Trial treatment costs*	These include the costs of the intervention itself, the purchase of any equipment associated with that intervention, and costs of continuing delivery of an intervention subsequent to the period of research. These may be sought either from industry, in the form of donation of drugs, equipment or funds or from NHS through their partnership arrangement with MRC and DH [2], or in exceptional circumstances as a specific request to DH. This latter option is available when an intervention has substantially greater costs to the NHS than the standard care which would normally be offered. Trial teams may apply for excess treatment costs to be met as a subvention by DH. The costs of placebos manufactured to match the active drugs are considered to be research costs but may be met by industry.

Even when funds have been secured, financial issues can be an ongoing concern for trialists, with problems arising during the course of a trial. One example is the effect of the need to comply with the recent EU Clinical Trials Directive; it has been argued that this places already strained budgets under additional pressures [7]. Some researchers have reported that these pressures have directly affected recruitment rates [8] and have in fact forced some trials to close [9,10].

How trial teams negotiate funding systems and how they address financial considerations which arise in the course of clinical trials has not yet been described in the research literature. There is in fact surprisingly little empirical work on financial considerations at all, other than brief references in a small number of USA-based studies [11-17]. These indicate the costs of conducting clinical research in terms of clinician hours may be considerable [11], and that a lack of funded clinician time for research [12-14], and a lack of support staff [15-17], are both thought to have a direct and negative impact upon recruitment rates [18]. In these few studies the financial focus largely relates to the accrual of patients into trials. There remains a lack of empirical evidence in relation to the subject of how trialists actually deal with financial considerations in their various forms and how these might shape the trials in which they are involved.

This paper reports data on this subject arising from a qualitative study which was carried out in 2004. It was conducted within the broader context of the Strategies for Trials Enrolment and Participation Study (STEPS), a multi-method research project which examined factors which might be positively or negatively associated with recruitment to RCTs [19,20]. STEPS was commissioned by the National Coordinating Centre for Research Methodology in the UK with funds from MRC and HTA. STEPS had three distinct components; an epidemiological review of a cohort of 114 trials funded by MRC and HTA [20]; the qualitative study which considered four trials which appeared to have particularly interesting lessons for recruitment [21]; single in-depth case study of a large multi-centre trial to examine the feasibility of applying a business-orientated analytical framework as a reference model in future trials [22].

The qualitative component of STEPS explored the views of UK trialists, clinicians and other professionals, all of whom were associated with one of four multi-centre trials. Two of the trials received funding from MRC and two from HTA. Although financial aspects of trial management were not the primary focus of this study, it became clear during data collection and analysis that they were of particular importance to many interviewees and were connected to a range of issues other than simply recruitment rates. The ways in which financial considerations were managed were often presented by the interviewees as crucial elements in the success of their trial. Finance was, however, also seen as a potentially problematic factor which could directly impact on aspects of the trial design and progress. The emergence of financial issues as an area of concern led to further consideration of this substantial strand of the data. This paper therefore describes the ways in which the four trial teams negotiated the UK funding system, and reports the views of informants on the impact of financial issues on the trials in question.

## Methods

### The research setting

In contrast to the approach often reported in the empirical literature, where research is conducted to understand why recruitment failed for a particular trial [15,23-27], the STEPS team wished to learn from trials which offered positive examples. The two funders of STEPS, MRC and HTA, were therefore asked to recommend a number of the trials in their research portfolios which they considered to be successful. After consultation between the funders and the STEPS team, four multicentre 'exemplar trials' were selected which had met, or were on schedule to meet, targets agreed with their funders. They were chosen to represent diverse research settings, methods and clinical specialties. One principal investigator (PI) declined participation as he intended to publish details of their successful recruitment practices separately. Another trial from the funders' lists was selected as a replacement.

The four trials considered here are: The Heart Protection Study (**HPS**) [28,29]; Trial of Chemotherapy for Bowel Cancer (Fluorouracil, Oxaliplatin and Irinotecan (CPT11) Use and Sequencing (**FOCUS**) [30]; A pragmatic single-blind RCT and health economic evaluation of leukotriene receptor antagonists in primary care at steps two and three of the National Asthma Guidelines (**ELEVATE**) [31]; and Trial of Outcome for Child & Adolescent Anorexia Nervosa (**TOuCAN**) [32]. Each trial was an ambitious project as, irrespective of the differences in their target sample sizes, each was intended to be the largest in the world for their particular population. Recruitment is now complete in all trials with targets exceeded for HPS and FOCUS, whilst TOuCAN, and ELEVATE exceeded their targets in the main elements of their samples. Broad details of these four trials are presented in Table 2 and their structure, history and progress are described in further detail elsewhere [19].

**Table 2:** The four trials

**TRIAL**	**SPECIALTY **	**POPULATION**	**MANAGEMENT **	**RECRUITMENT SETTING**	**INTERVENTIONS**	**DESIGN**	**FUNDERS**
**HPS** (The **H**eart **P**rotection **S**tudy)	Cardiovascular health promotion	Patients at increased risk of coronary heart disease	Clinical Trials Unit	Hospital	- Statins (Simvastatin) -Antioxidant vitamins - Placebo	4 arm randomised trial (2 × 2 factorial)	MRC BHF Industry
**FOCUS** (Trial of Chemotherapy for Bowel Cancer (**F**luorouracil, **O**xaliplatin and Irinotecan (**C**PT11), **U**se and **S**equencing)	Oncology	Patients with advanced metastatic colorectal cancer	Clinical Trials Unit	Hospital	Chemotherapeutic agents - Modified de gramont with fluorouracil - Irinotecan - Oxaliplatin	5 arm randomised trial with cross-over	MRC Industry NHS Trusts
**ELEVATE** (A pragmatic single-blind RCT and health economic evaluation of leukotriene receptor antagonists in primary care at steps two and three of the National Asthma Guidelines)	Asthma management	Primary care patients with asthma requiring regular preventative treatment or an increase in therapy	University Department	General Practice	- Leukotriene receptor antagonists - Inhaled corticosteroids - long-acting beta agonists	3 arm randomised trial (with option to switch treatments post-randomisation and parallel non-randomised 'naturalistic' cohort for patients refusing randomisation)	HTA Industry PCTs
**TOuCAN** (**T**rial of **O**utcome for **C**hild &**A**dolescent Anorexia **N**ervosa)	Adolescent psychiatry	Adolescents (aged 12–18) with anorexia nervosa	Hospital/university	Hospital and out-patient service	- intensive inpatient treatment - general local outpatient service - specialist outpatient service	2 severity strata (steps) with 2 arms at each step	HTA

### Ethics

Multi-centre Research Ethics Committee approval was given for the qualitative study under "no local researcher" guidelines. The study conformed to local research governance requirements and approval was given by 12 NHS Trusts Research and Development departments.

#### Sampling and recruitment process

Despite the fact that many different professional groups contribute to running a clinical trial, there is little research which recognises the variety of roles involved. Exceptions are the work by May and colleagues which considers the evaluation of telehealth interventions from the perspective of clinicians, technical experts, evaluators, managers, policy actors and patients [33,34] and by Hamilton-Brown which considers the tension between research and clinical practice in the area of substance abuse treatment for clinicians and research staff [35]. The research samples which are available predominantly seek the views of senior doctors, largely those associated with oncology trials [24,36-43]. There are some studies which include nurses associated with trials [15,44] and general practitioner (GP) recruiters [45-48]. We were unable to identify research which considered the views of trial co-ordinating staff.

For this study a broad model of potential interviewees was drawn up at the planning phase, based upon an initial concept of which professional groups were likely to contribute to running the trials. The study was designed to include 32 interviews, 8 per trial, split between central co-ordinating staff and staff from recruiting clinical centres. The intended interviewees were PIs, trial managers, local lead consultants in the recruiting centres, and local recruiters (doctors or nurses depending on trial procedures). The aim of representing this range of responsibilities was not to characterise the views of particular professional groups, but to draw upon and explore different experiences of the four trials. By allowing these key players to describe the history and workings of each trial, multiple perspectives could be used to generate a composite and nuanced account of how the trials developed and were run.

As understanding of the processes involved in each trial increased, a flexible and responsive approach to recruitment was adopted. The list of interviewees was adapted and expanded accordingly to fit the unique circumstances of each trial. The first interviewees were recruited through purposive selection methods. The funders, MRC and HTA, introduced the study team to the PIs, who in turn facilitated access to the central trial teams and some recruiting centre staff. The central teams made contact with a range of collaborators via their own mailing lists. Those interested in taking part in the research were invited to respond directly to the STEPS researchers to preserve confidentiality. Once interviews were underway recruitment procedures drew upon elements of snowball sampling as interviewees suggested the importance of approaching particular colleagues with specific roles in a trial. In a small number of cases the researcher made an unmediated direct approach to individuals who were detailed in the trial literature who seemed to be potentially important to the study. Given the combination of direct and mediated approaches to some individuals, an opt-in volunteer system for others, and the fact that it is not known how many individuals were contacted by the trial teams, it is not possible to give a response rate. This approach to recruitment added 13 interviews to the study, and the sample comprised 45 interviews in total (Table 3).

**Table 3:** Sample structure

	**FOCUS**	**ELEVATE**	**TOuCAN**	**HPS**	**TOTALS**
**CENTRAL CO-ORDINATING STAFF**					

PIs	√*	√*√*	√*√*	√*	**6**
Trial managers	√	√*	√*	√*√*	**5**
Central recruiters		√√	√√		**4**
Administrative support		√			**1**
Statistician	√*				**1**
Clinical support	√*		√*√	√	**4**
	4	6	7	4	**21**

**CLINICAL STAFF IN RECRUITING/REFERRING CENTRES**					

Local lead investigators	√*√*	√*√√	√*√*√*√√√	√*√	**13**
Recruiting doctors	√√				**2**
Recruiting nurses	√√*√*	√*√√		√√√	**9**
	7	6	6	5	**24**
**TOTAL STAFF**	**11**	**12**	**13**	**9**	**45**
* CITED HERE	**7**	**5**	**7**	**4**	**23**

At the end of the study 11 interviewees (5 PIs, 4 trial managers, 1 local lead and 1 academic staff) were contacted again for validation purposes. They were asked to view an almost final draft of the relevant chapter in the report to the STEPS commissioners, as well as this paper, to monitor accuracy and to verify the findings. Small amendments to factual details were suggested and all supported the conclusions that were drawn.

### The interviews

All interviews were carried out by telephone between December 2003 and May 2004. They were tape-recorded with the consent of interviewees and fully transcribed. They were semi-structured and typically took between 20 and 80 minutes, depending upon the degree of connection with a trial. Discussions were often wide ranging. They highlighted very different levels and types of responsibilities, as well as individual attitudes to, and experiences of, the trials. Because the study included professionals with a variety of roles, some questions varied from interview to interview, as well as from trial to trial. The lines of questioning were also modified and developed during fieldwork, in response to the insights gained. To preserve confidentiality, interviewees are not named. Their trial-specific role is not given unless unlinked from a specific trial. They are identified by their interview number preceded by the first initial of the trial acronym e.g. H-1.

This study used telephone interviews to collect qualitative data. The possible advantages and disadvantages of this method have been considered by several authors [49-51]. Telephone interviews are often used in conjunction with other approaches in mixed methods studies [52-54], but have also been used as a single data collection method with patients [55,56], and clinicians [57,58]. Sweet [59] describes this approach as "a methodologically and economically valuable data collection technique in qualitative research" (p.58). In this study the use of telephone interviews permitted recruitment of a sample with a wider geographic distribution (England, Scotland and Wales) than would have otherwise have been possible. It allowed the team to respond to the emergent need to include additional interviewees. The topic under consideration was amenable to discussion by telephone and there was no sense that the discussions or the researchers' understanding of the data were limited as a result of this method.

### Analysis

All interviews were conducted, read and analysed by one researcher (CS), with team members (JG and DE) reading a sample of interview transcripts, and commenting on significant portions of data as requested. Data analysis was conducted with the assistance of the qualitative package Atlas-ti. The analysis was shaped by the intention to provide data for each trial which were descriptive, exploratory and comparative in nature and took direction from Layder's "adaptive theory" [60]. Layder suggests an approach whereby pre-existing researcher knowledge and concepts are an explicit part of the analytic process. Existing models held by the researchers are adapted in a process of modification and refinement as experience and understanding of a phenomenon grows. The study findings were therefore a product of both pre-existing perceptions of what was likely to be of importance (the interview schedule) and the unanticipated issues which were introduced and made clear through the accounts of the interviewees themselves. The significance of financial considerations was the most striking of the issues which emerged from the interview and analytic processes.

Financial issues were originally considered within the larger interview schedule through the practical process of setting up a trial and whether or not a funded extension was later required. As the interviews progressed increasing attention was paid to the ways in which trial teams dealt with financial matters and the impact they might have on their research. For the qualitative study as a whole, which considered factors which may have contributed to successful recruitment records, the analytic process identified four overlapping Key Stages in the course of each trial; these were "Foundation work" "Recruitment", "Delivery of care" and "Delivery of Research". Foundation work included engagement of scientific and clinical collaborators, establishment and communication of a trial's scientific credentials through development of appropriate research questions and methods, and attention to funding and financial considerations.

Further analysis of the interviews where financial considerations were discussed, highlighted and drew out the ongoing and mutable nature of this element of the trials process. Twenty-three interviewees are cited here (HPS 4, FOCUS 7, TOuCAN 7, ELEVATE 5). Financial considerations clearly cut across all of the Key Stages. Recognition of the way they are interwoven throughout the course of the trials led to delineation of three broad areas of financial activity, namely:

• Economic foundation work

• Maintenance work

• Resourcing completion

The division of different types of costs associated with clinical research in the UK, and our division of financial considerations into three broad areas, might suggest a neat, compartmentalised approach to funding. They imply discrete and definable costs which would be met through a clear budget at the foundation stage, before research gets underway. In fact the interviewees described a fluid response to the funding system and reported a variety of funding-related experiences. The similarities and differences between the ways in which financial considerations were experienced in each of the four trials are considered in detail below

## Results

### Economic foundation work

For many trials the preparatory work to develop the intellectual direction of the research, to gain the trust and support of potential clinical collaborators, to attract the interest of funders, and to address logistical considerations, starts several years before a trial actually commences. This process can involve a long run-in period of discussion with a variety of potential funders, to consider the value of the proposed research, its design, and to plan financial support for costly trial interventions.

#### Negotiations with industry for treatment costs

For two of the trials, HPS and FOCUS, the preparatory process included negotiations with representatives from the pharmaceutical industry from a very early stage.

The funding strategy employed by the HPS team initially relied exclusively on industry to fully support the costs of both their pilot study and their main trial. The team aimed to evaluate cholesterol-lowering therapy (statins) and antioxidant vitamins for a diverse group of patients at increased risk of coronary heart disease. The very large quantities of active drugs, placebos and vitamins which were to be used represented a considerable cost. In the course of the industry-funded pilot work the trialists realised that a major shift in study design was needed in order to best answer the research questions and a decision was made to substantially increase the sample size. The associated rise in costs caused difficulties in negotiations with the intended funders, and brought about a long delay between the pilot and the main trial, as explained by a member of the central trial team:

*The gap that occurred between the pilot and the main study was merely an issue of getting the funding. We had initially been thinking about a ten thousand patient study but the more we got into it the more we felt that a bigger study of twenty thousand patients would make more sense and we couldn't persuade [the drug company] to fund that*. (H-40)

If the trialists wished to maintain control of the research design and to increase their sample size, they had to forgo their initial funding arrangement. With such a large and hugely expensive trial in mind, they faced an enormous financial challenge. It was necessary to bring together a consortium of funders, each to be responsible for a different element of the trial costs. This resulted in a highly complicated seven year process in which there were several offers and retractions of offers of funds. Another member of the central trial team explained that in this period their funding situation was extremely precarious, with a delicate dynamic between potential funders: "people wouldn't want to commit unless other people had committed and so ... very skilful negotiations had to take place" (H-42). The trial eventually cost £21 million. Research costs were shared by MRC and the British Heart Foundation. Two major pharmaceutical companies met the substantial treatment costs associated with their own products. Securing and co-ordinating this level of multi-sourced funding was "extremely difficult" (H-40), and required "a prolonged effort" (H-39). The lag between the pilot study and the main trial proved to be of consequence; it not only delayed publication of useful research findings, it also allowed other trials of cholesterol-lowering therapies to report their results during the intervention period for HPS. Some clinicians associated with HPS altered their treatment patterns in response and it was thought that this may have diluted the size of the effect of the trial interventions in HPS. One interviewee explained:

There were some types of patients in HPS for whom their own doctors [decided] to use cholesterol-lowering therapy. The study would have achieved a bigger LDL [low-density lipoprotein] cholesterol difference between the treatment groups if it had been done earlier. (H-40)

For HPS the size of the contribution required from industry was the trigger for renegotiation of their funding arrangements. For the FOCUS trial, additional considerations shaped their discussion with potential funders. The history of the development of this trial was described in a small number of interviews which revealed a similarly difficult period of complex discussion and negotiation with industry.

FOCUS was designed to address the issue of how best to treat patients with advanced metastatic colorectal cancer, in the light of the development of two chemotherapeutic drugs, irinotecan and oxaliplatin. These drugs had been evaluated in previous trials but had not been directly compared, nor had there been direct comparison of the same drug used in first- or second-line therapy. One of the interviewees explained that they felt that an assessment of these drugs by "the independent academic sector" was important."

*There were a number of issues which we felt needed addressing which were, if you like, different in nature to the type of questions which were being posed in the drug company trials using those compounds. And we felt that there was a need ... for a large trial which had end points which were relevant to patient care, looking at overall survival over the duration of treatment and also looking at issues for quality of life and toxicity when those new drugs were being used*. (F-2)

The FOCUS team faced financial, logistical and political challenges given the very high cost of these drugs, the inclusion of NHS patients, and the potential differences in the academic and industry research agenda. A particular difficulty lay in the fact that they planned to "compare drugs [from the two different companies] head to head, and drug companies are very unwilling to do that normally" (F-4). Furthermore, the team were trying to establish the foundations of their research in a particularly difficult system, as an interviewee explained.

[T]he NHS was not well set up for doing research involving expensive drugs unless [a] drug company came along and provided those drugs. ... [T]he dilemma we had with FOCUS was asking questions which were independent of the drug companies and weren't necessarily commercial questions which the drug companies wanted [us] to ask, but at the same time using their drugs, which were expensive. (F-2)

The challenge they faced was "how to obtain the drugs and get them into a trial which would involve a lot of patients in NHS hospitals around the country" (F-4).

The interviewees described what one termed as "a long drawn out process of negotiation" (F-2) with industry as well as with the NHS (see below) to bring together the level of funding needed for the treatment costs. The descriptions of this process made it clear that in this period of trying to reach agreements over funding, the commercial agenda has the potential to influence the design of a developing trial. The interviewee quoted above argued that this agenda is of great importance, stating "they don't want to do research which is going to put them out of business" (F-2).

*When you negotiate with drug companies over obtaining drug supplies for trials, you usually find yourself negotiating with somebody who's from the business unit or marketing unit of the company where, obviously, the priorities and the issues which they bring to bear when they're looking at trial designs and trying to make decisions about whether they're going to invest will be commercial and business decisions*. (F-2)

The process by which the trial team and the drug companies came to their agreement about funding and research design was "fairly tortuous and painful at times" (F-3). An interviewee described how this tricky and delicate process can be facilitated or inhibited by some of the less formal elements of the negotiations.

*What happens is that individual clinicians ... know individual people in drug companies, and there are sort of contacts made. And sometimes ... you have very good rapport with those people and they can sort of oil the wheels and make things happen, and other times it's very difficult. ... We had someone who was very supportive of the trial in one of the drug companies, and they really pushed it very hard with ... whoever makes decisions ... but in the other drug company, there was someone who ... just wasn't willing to play ball at all *... (F-4)

Over a two year period in which, as for HPS, offers of funds were made, modified, withdrawn and renegotiated, the team had to try to "maintain our integrity and not to compromise the questions and the integrity of the trial design" (F-2). Key informants felt that despite the difficulties they faced, they had not made concessions over the research question or trial design, one commenting that " [we] held our ground" (F-4. This was not, however, without financial implications.

*We ended up with a trial design where we had pretty much stuck to our guns and where the amount of drug company funding was very much less than we might have [had] if we had compromised the design a bit more*. (F-2)

With hindsight it was suggested that the difficult negotiations, and the multiple offers and retraction of offers of funds, had created significant and inappropriate delays to the research. This led to reflection on the practice of involving industry when research questions are of importance to the NHS but are not of direct commercial interest.

In retrospect it might've been better if we hadn't actually obtained any funding from drug companies in the first place for FOCUS and we'd actually gone along to the subvention committee with a more expensive trial and said ... "We want to do this trial. We've got no support from drug companies, please provide a subvention" and maybe they would've done that. (F-2)

#### Seeking research costs from public sector funding bodies

Public sector funders have an interest in ensuring that the research they support asks appropriate questions of importance for public health and public services in a scientifically rigorous way. When they apply for funds, trialists can be required to defend or modify their proposals in order to meet the standards set by the funding bodies.

The four trials considered here were all successful in their applications for research funds from MRC or HTA. For FOCUS, this stage appeared to be the least problematic element of their difficult funding process. A member of the central trial team commented that their research costs were fully met and their application to MRC "went through without a hitch." (F-4).

HPS also received funds from MRC and the importance of prior negotiations between the researchers and the funder was discussed in one interview. The trial was designed to include, amongst other subgroups, older patients and those with already low cholesterol levels, as little was known about the impact of reducing cholesterol for these groups. These suggestions were of concern to MRC, and were also raised in their discussions with industry. MRC wished to impose a lower upper age limit, and a higher lowest cholesterol level on the eligibility criteria, largely from concern that there were no precedents for lowering cholesterol for these groups. The researchers and MRC came to an agreement:

*[O]ur protocol initially had no upper age limit and no lower cholesterol level and .. particularly MRC said they would not fund us if we didn't have an upper age limit and we didn't have a lower cholesterol level. [W]ith respect to the lower cholesterol level we managed to get agreement that it be at a level that would actually exclude no one or almost no one... [For age limit] we could shift the upper age limit up if the Data Monitoring Committee on reviewing ... the first thousand patients or so... did not observe any obvious problems and so ... we then shifted it up but .. it was difficult at that stage to include very much older patients*. (H-40)

In comparison to the lengthy developmental processes for FOCUS and HPS, ELEVATE and TOuCAN were designed in response to HTA calls for applications to address very tightly defined research questions. For ELEVATE, the call was fortuitous as some of the senior trialists were already wishing to address the same issues given their own research interests. The trial that they developed compared the role and cost of a class of orally administered drugs, leukotriene receptor antagonists (LTRAs), to inhaled corticosteroids (ICS) and long-acting beta agonist (LABs) for primary care patients who have asthma which requires regular preventative treatment or an increase in therapy. LTRAs have been available for some years and can be prescribed for asthma patients but LABs are more commonly used. LTRAs are of interest as they are in tablet form and may prove to be more acceptable than drugs delivered via inhalers, and so may promote better disease control. They are however the more expensive intervention. A member of the ELEVATE team explained that the call to consider this area was to some extent a coincidence, but it was also a reflection of the direction that research in the area was taking. It "fitted with what we were wanting to do. ... We had recognised it was a needed research area and also HTA had." (E-15). For this application "the bidding group ... fell together very easily and looked like a strong team." (E-13). The team had to work together quickly to develop their application.

*We fought and negotiated and struggled over a protocol that one, met our research agenda and two, met the research agenda of HTA, to come up with a protocol that was real-life, going to give us cost-effectiveness data, [and] was do-able in general practice ... And then got through to the second round at which time you then spend a lot more time. ... [Y]ou get a little bit of feedback. I guess the one bit of feedback that in retrospect was probably a problem was from HTA that they thought our costs for general practice were too expensive. ... Reviewers often do not understand how difficult it is to truly deliver research in primary care*. (E-15)

The team altered their plan to train audit assistants from general practice to assist with the trial, and replaced this with a cheaper option to employ a research assistant, despite feeling that they had suggested realistic costs. This cost-based decision had later ramifications for the recruitment process (see below). An interviewee who had supported the initial approach to recruitment commented that in retrospect he felt they possibly "should have stuck more firmly to [our] guns knowing what I do now. But we did that and ... we submitted a full protocol ... and got accepted." (E-15). The team then experienced a delay related to a general HTA funding issue in which the future of ELEVATE was unclear; they felt that HTA was going to "pull the funding for this project" (E-15). In the words of another interviewee, the trial "sat on the shelf for about a year and a half" (E-12) before they were told that it could proceed. Negotiations for the treatment and excess treatment costs for ELEVATE were started after the initial research funds were awarded.

As for ELEVATE, the senior TOuCAN researchers had wished to carry out a trial in their specific area for some time. They wished to assess the efficacy of approaches to care for adolescents with anorexia nervosa and were keen to understand whether it was better to have intensive inpatient treatment at an earlier stage in the condition, or whether this should be reserved for the more difficult, entrenched cases. They also wished to assess the possible value of a recently developed specialist outpatient service. Whilst the ELEVATE team felt that there was a degree of coincidence in their own interests and those of HTA, an interviewee from the TOuCAN team felt that the clinicians in their area had been proactive in raising the issue of the need for research and that HTA, after a period of consultation, had responded:

*[I]t comes about by clinicians and researchers ... lobbying or pushing ... for the need for it and eventually the message being heard by funders*. (T-27)

RCTs are not widely used in relation to the management and treatment of anorexia nervosa [61] and previous attempts to bring about a trial had been unsuccessful. The HTA call for proposals was not specifically for a randomised trial, but several interviewees felt that the radical step of suggesting this method was important in the success of their application. Cost was not mentioned as an element in the funding decision for this trial.

#### Research costs relating to staffing decisions

Within discussion of research costs more generally, a particularly important sub-category of funding issues emerged. The funds awarded by MRC and HTA cover a range of costs associated with running research, but the interviewees predominantly focussed on those associated with the employment of research staff. Whilst other costs such as institutional overheads or computing resources are generally fixed or reflect straightforward costing decisions, those associated with staff stem directly from practical and strategic decisions about how individuals will be deployed to manage and facilitate a trial. They relate to matters of research design (e.g. number of intended participants, number of collaborating centres), and to administrative and recruitment strategies (location of recruitment, responsibility for identification and recruitment procedures). Although funders do not make their decisions based purely on the total cost of a proposal, salaries do add substantially to overall running costs and decisions about staffing could directly affect the chance of funding. Proposals which are seen as inappropriately expensive, or those where costs have been trimmed to the extent that the research is not feasible, are both likely to encounter difficulties.

Each trial team chose to staff their research and delineate responsibility for the identification and recruitment of research participants in different ways. The choices made suggested that two elements underpinned their approaches. These were, firstly, the strategic use of available resources, and secondly, recognition of the need to take account of the specific conditions of each clinical setting.

The trial which undoubtedly placed the greatest investment in staff was HPS, a long-term trial with 69 recruiting centres, each with at least one fully-funded, centrally-trained, research nurse. The research nurses were not involved in the processes of identifying eligible patients. They ran trial-dedicated clinics in collaborating hospitals to which potentially eligible patients were invited. The identification and invitation process was controlled by the central administrative team. The research nurse assessed eligibility, discussed trial enrolment and recruited trial participants. A member of the central co-ordination team explained that this very specific demarcation of roles was crucial to keep a large multi-centre trial on track and to keep substantial costs under control:

*It is a big study to keep the budget under control. We needed to think of ways to keep the cost down and we felt that if ... the processing of the data, the invitations, the appointments, all the administration was central rather than peripheral then that should be another way of increasing efficiency and reducing costs*. (H-40)

A clinician who acted as the local lead investigator in a collaborating centre affirmed the value of the use of staff funded directly by the trial in practical and political terms. He was asked whether it would have been feasible to have collaborated with the trial without such a post.

*Oh no, no, no, absolutely not! I think for research like this it has to be funded separately. If it was in competition with our service commitment it would very quickly be sidelined and we wouldn't be able to do it*. (H-44)

Although the resources needed to employ staff to carry out recruitment are legitimate research costs, they were not always included within the initial request for research funds. This was the case for TOuCAN which utilised a very different staffing structure and recruitment strategy from HPS. The trialists were aware that recruitment for TOuCAN was potentially very difficult; there was little precedent for RCTs in relation to the management of eating disorders, the potential participants were widely distributed across the caseloads of many clinicians in the region, and it was important to gain access to a high proportion of these cases. They did not, however, feel that employing a large number of trial staff to control the process of identifying potential participants would be helpful. TOuCAN was deliberately and firmly set within existing forms of care, and involved a network of consultant psychiatrists in a highly localised regional setting in the recruitment process. The trial was "embedded in the [local] clinical service" (T-33) and much effort was directed towards generating and maintaining support for the research, with "lots of persuasion, lots of visits to local services" (T-27) with an emphasis on "building relationships with the consultants" (T-30). One consultant acknowledged the work that was done to "keep people like me on side" (T-32). The success of the first phase of recruitment rested entirely on the ongoing identification of eligible patients by the collaborating clinicians as each was referred into their caseload. This crucial role was unpaid and voluntary. The subsequent administrative and recruitment processes were controlled by a very small number of central trial staff whose salaries were met via the HTA research funds

Although there was little in terms of financial incentive to collaborate with the trial, some collaborators felt that there were potentially beneficial resource implications. Half of those enrolled in TOuCAN would be randomized away from the care of their referring clinician, and effectively moved on to the caseload of the specialist service. Whilst it was said that some clinicians felt a degree of concern over losing control of these very sensitive cases, it was also argued that it could be very helpful if some of these individuals, who can be "costly cases in terms of time and resources" (T-38) would be passed over to receive specialist care, and that it can be "a relief to get a difficult case off your mind" (T-35).

The system of relying on the goodwill and interest of the collaborating clinicians for referral worked well, and the trial accessed the majority of the target cases. This appeared to be precisely because it was so closely backed onto existing professional and clinical systems of referral that according to a referring clinician it "just sort of blended in with what you'd usually do." (T-38). Another clinician argued however that this was due to the very particular nature of TOuCAN, and that other trials would require a different type of research support from a central trial team:

*[W]hen we've been involved in trials before of things that are commoner, and where the burden on the service of actually referring or involving people in trials is more, ... in those circumstances providing additional administrative resources as well as nursing resources would be a really important factor. ... [W]here we've been involved in research, it's been the admin team which is really overwhelmed by extra demands.... . [For trials] in ADHD genetics and depression treatments and things like that, we would've needed both nursing support or, you know, some kind of clinical support and admin support to be able to do these things easily*. (T-32)

The decisions about how to fund the staff associated with the FOCUS trial were quite different from those made for the other trials. Clinical research in oncology is overseen and supported by the National Cancer Research Network (NCRN). Research is high profile and collaboration is routine in most large oncology centres. Specialised research nurses are essential members of staff who work with whichever trials are linked to their unit. The FOCUS Trial was developed as part of a rolling programme of research and drew on the support of an existing network of committed collaborators. This determined both the staffing structure for the trial and the associated financial costs.

Procedures for identification and recruitment of participants were entirely under the control of experienced staff whose salaries were already funded in the collaborating centres. As for TOuCAN, it was usual for a consultant to identify eligible patients as they presented in their clinical caseload. The consultant would initially explain the trial to patients, usually in the context of discussion of the management of their advancing metastatic disease. They would be referred on to a research nurse who would give further details of FOCUS and arrange to see the patient again. At the subsequent appointment the nurse would go through consent procedures if they wished to enrol in the trial and initiate the allocated intervention. In this way the clinical staff worked in close collaboration with research-related staff.

The collaborating centres were given a contribution towards the costs of their research nurses from the FOCUS research funds. This allowed for some protected time for FOCUS, but much of the cost associated with recruitment and subsequent administrative tasks were in fact absorbed by the centres. This was possible because they had their own resources and staffing systems developed specifically to support research collaboration. Interviewees from one recruiting centre explained that resources for a research nurse who could work with FOCUS and other oncology trials were provided through Trust R&D funding systems. Another interviewee explained how their ability to collaborate with research had increased as a result of the ability to apply for their own funding for research-related staff from the NCRN. This was seen as a positive step as it permitted centres to raise their level of collaboration with trials more generally:

*[W]ith the NCRN, we've got 12 nurses now and some data managers so it actually does make it easier... There is a definite improvement*. (F-6)

Many oncology centres collaborate with industry-sponsored trials and it is common for payments to be made for each participant recruited. An academic member of the FOCUS team suggested that these payments, which can be "in thousands" (F-1), are "not at all a bad thing" precisely because they can provide an element of stability to the research infrastructure within collaborating centres. Effectively the costs of collaboration with trials such as FOCUS are subsidised though existing links with industry. In comparison to the payments made to recruiting centres by industry trials, those from public sector trials can be far less lucrative. A senior research nurse explained how the less lucrative links can be problematic.

*[W]e find that the money we're gaining from commercially sponsored studies actually pays for the nurses. ... [T]he NRCN studies only give us something like two hundred pounds a patient for data collection. ... It costs a lot more than that, and in our institution it's usually the industry-sponsored studies, the money we make from that [which] will pay for the nurses. ... We have a commitment to MRC trials first. We would always recruit to them before an industry- sponsored one because we're an academic department. [But there are] ... issues of paying research nurses' wages and [if] you've got a study that pays five thousand pounds per patient or one that pays two hundred pounds per patient.........! *(F-10)

An interview with another research nurse suggested that in collaborating centres, competition for staff time between non-industry and industry-led trials can also be problematic. The nurse argued that "one of the main problems was staffing" (F-9). Centres can be under "a lot of pressure from the drug companies" (F-9) to maintain their records and keep on top of their trial-related administration, wary of the possibility of inspection of records in relation to the EU Directive. This pressure meant that in this centre efforts were diverted from the public sector trials which therefore fell behind; essentially "other priorities had come into the forefront" (F-9). A research nurse post was developed by local staff to address an administrative trial-related backlog in their centre.

*FOCUS ... had sort of fallen by-the-by with data management ... [T]he sheer workload was too much for the research nurses that we had*. (F-9)

While attempt were made to catch up with the administrative backlog for FOCUS, clinicians put recruitment of new patients temporarily on hold.

*[F]or a couple of months [they] just put people on the standard [treatment] and didn't [recruit] because [they] knew that we couldn't manage the workload*. (F-9)

This account suggested important ways in which financial issues, in practical and political terms, directly shaped recruitment to a trial at a local, behind-the-scenes, level.

#### Negotiations with service providers in relation to NHS support costs and excess treatment costs

With research costs covered by MRC or HTA, and the costs, or partial costs, of drugs to be used in a trial secured from an external source, the trialists also had to consider how to meet any excess costs associated with unfunded drugs, and the delivery and assessment of care. As HPS had agreed the funds for the trial drugs with industry, and removed the delivery of the research interventions and subsequent assessment from the NHS setting, there was no obligation to negotiate NHS support costs or excess treatment costs. The interventions in TOuCAN so closely approximated the existing care arrangements that again there was not an issue of excess costs to the NHS. By contrast the negotiations for FOCUS and ELEVATE were complicated and sensitive.

Trials such as FOCUS which have MRC funding should, in theory, be supported by an agreement with NHS Trusts, known as the NHS Research Concordat [2,61]. Under the terms of this agreement, if MRC covers research costs, NHS Trusts are obliged to take responsibility for 'legitimate' service support and treatment costs associated with a trial. It was anticipated that the Concordat would facilitate collaboration with FOCUS for individual centres and generally the NHS Trusts did comply with the Concordat. The Trusts were, however, "in different states of financial distress around the country" (F-2), and the excess treatment costs associated with the trial were substantial, and the agreement did not provide the broad support that had been expected. It was necessary to apply to the NHS Trust of each potential collaborating centre, for the excess treatment costs to be met. One of the clinical lead investigators in a collaborating centre explained how for them it had been "straightforward", but was not so for others.

*[In our region the Concordat is] considered to be authoritative and [they] will honour their responsibilities to provide us with excess treatment costs. ... I'm very aware of other centres and the problems they had [when their Trust] would not fund the excess treatment costs in this trial. So a number of areas ... were not able to contribute patients, although they wanted to*. (F-3)

The ELEVATE trialists faced a similar issue in that GP practices which might collaborate with their trial were accountable to NHS Primary Care Trusts (PCTs) for any increase in their prescribing budgets incurred through their association with the trial. In this setting there is no equivalent to the Concordat. Rather than relying on PCTs to, in effect, subsidise the research, the trialists entered negotiations which centred on their keeping costs to the PCTs "neutral" (E-15). The trialists provided funds to the PCTs to offset any treatment costs and so facilitate their collaboration and that of the GP practices. These offsetting funds were sought from industry. One interviewee explained the importance of this.

*We managed to get industry to make a ... contribution to the excess treatment costs, to head off the resistance we thought we might encounter particularly from the [PCTs] holding the drug-prescribing budgets for primary care. ... Because the leukotriene receptor antagonists are more expensive than other therapies, it could have pushed up their prescribing costs. ... Certainly once you make a formal approach to GPs to participate in a study like this, one of the questions that they are bound to ask is 'Is this going to get us into trouble with the PCT?' ... So we managed to get a no-strings-attached contribution from the two companies who manufacture leukotriene receptor antagonists, which we've been able to pay into the local PCT drug budgets, on a proportionate basis according to the number of practices that were participating in the study. ... I think if we hadn't been able to get that going, we might have had whole PCTs [and] whole groups of practices who might have said 'No, we won't participate because the excess treatments costs aren't covered'*. (E-13)

The practices that collaborated with the trial were also given a contribution to their funds to cover nursing time, something which was thought to be likely to promote "a stronger collaboration" (E-13). Although one GP argued that this did not in fact cover the costs involved, it was still widely viewed as an effective and essential strategy.

### Maintenance work

The bulk of the financial considerations for these trials undoubtedly occurred in the foundation stages described above. It became apparent, however, that even when the trials were funded and recruitment was underway, financial challenges could arise at further points in the course of the research. This was particularly evident for two of the trials, ELEVATE and FOCUS, where trialists had to respond and adapt to changing financial situations in two particular areas. Interviewees from these two trials described:

• the need to renegotiate trial finances

• the need to weather financial storms

#### The need to renegotiate trial finances

ELEVATE encountered major difficulties in recruitment and this was accounted for partly by the 18 month delay in receiving the funds from HTA. The time-lag meant that the trialists were faced with carrying out their research in a different research climate from that which they had envisaged. The trial involved recruitment of patients with uncontrolled asthma, who would be appropriate candidates for a change in their therapy. Interest in this group of patients had grown since the funds were initially awarded.

*[P]harma-companies were really pushing for this group of patients to go on to additional therapy so many of our eligible patients had been 'stolen' if you like by the pharmaceutical companies. ... I mean there was still a pool of patients there but they were the slightly more difficult-to-access patients*. (E-15)

This background of competition with an industry trial for eligible patients was undoubtedly a challenge, and was exacerbated by difficulties encountered in the general practice environment. ELEVATE, like TOuCAN, aimed to bed the research into the clinical setting by including GP practices in recruitment processes. As the trial was developed by GPs, in consultation with GPs, there was sensitivity to the fact that costs which might be incurred could have personal as well as institutional financial implications for collaborators. A GP explained the nature of the problem:

*General practices are actually small businesses and they are funded in a completely different way to hospitals. ... If you are a teaching hospital there may be funding in the system for the idea of supporting research. In general practice research is not a core thing that is funded at all. ... Even things like sending out letters, stamps on envelopes and secretarial time, all of that costs money. The only place that that money will come out of is the practice profits, which is basically the partners' income. So unless it is adequately funded you are actually asking the individual GPs to take home less money in order to do the research. That is just not the case in hospitals. It doesn't affect the doctors' or nurses' incomes at all [if] research goes on*. (E-23)

Although the trial team were aware of these issues, the trial still required greater human resources than were anticipated. It quickly became evident that recruitment problems in the GP practices were, in great part due to the trial-related workload which fell to the practice staff. The trial team reacted swiftly, seeking additional research funds from HTA in order to radically modify their administrative procedures. The entire method of identification and recruitment of potential participants was refined and shifted towards an approach which was similar to that used by HPS. The renegotiated HTA funds allowed the workload to be directed away from practices, removing the requirement for their staff to carry out time-consuming searches to identify eligible patients, through payment for additional trial staff and the occasional use of independent agency research nurses. GPs and practice nurses mentioned during the interviews that without this more intensive input, collaboration would have been difficult if not impossible.

*The most important thing in the ELEVATE Study has been the support from the researchers. ... I contrast that with a study I was doing for a commercial research organisation which was overly burdensome with paperwork, very poor support from the research assistants ... and in the end we didn't recruit anybody for their trial*. (E-21)

#### The need to weather financial storms

For ELEVATE the problems with recruitment were identified early in the course of the trial. For FOCUS, difficulties arose in relation to debates about the evidence of clinical effectiveness and treatment costs which could have derailed the trial, two years into recruitment. In 2002 the National Institute for Clinical Excellence (NICE) argued that existing and emerging data on improvements in survival times for irinotecan and oxaliplatin did not warrant the costs associated with their routine use in first-line therapy. NICE issued guidelines recommending that irinotecan should be used only after failure of a first-line treatment, and that the use of oxaliplatin be restricted to a small sub-category of patients [62]. The guidelines affected FOCUS in several ways. Firstly, except for a subgroup, oxaliplatin was now available only via the trial, to which NICE recommended recruitment. Secondly, NICE had recommended second-line irinotecan when first-line treatments had failed, when two of the arms in the trial did not employ irinotecan. Furthermore, NHS Trusts which had not permitted their hospital departments to refer to FOCUS because of the associated treatment costs, were being specifically directed to make such referrals through the NICE guidance.

The NICE guidance caused concerns which were expressed in a letter to a newspaper by 28 oncologists, almost half of the UK oncologists practising in this speciality. They argued that the restrictions placed around irinotecan and oxaliplatin would reduce life expectancy [63]. The trial team responded in this difficult period by adapting the trial design to allow patients who had received oxaliplatin to cross over to irinotecan, and those that had received irinotecan to cross over to oxaliplatin. At this stage the pharmaceutical company withdrew its support and stopped providing irinotecan for FOCUS, a move which interviewees felt was a direct consequence of NICE's guidelines on treatment costs. This appeared to be a major financial challenge to the trial, with several interviewees stating that they expected serious consequences, "an absolute disaster" (F-4). In spite of these concerns, the recruitment rate remained steady, with the collaborators' NHS Trusts effectively absorbing the treatment costs for patients who were referred to the trial.

Information gleaned from interviewees from the collaborating centres sheds further light on possible reasons for the continuing commitment to the trial. Although it was clear that the costs of the drugs, and the limitations imposed by NICE did cause concern, some stated that after the NICE guidance they recruited to the trial with the explicit aim of accessing the drugs. A consultant described how he presented the complicated funding situation for oncology drugs to patients who were eligible for FOCUS. He described a need to balance giving honest information without applying pressure, given the already difficult situation of those with advanced metastatic cancer:

*You don't want to force a patient to go into trial, but I do say that by going into the FOCUS study, there is oxaliplatin available. We don't have funding for that outside the study*. (F-6)

It seemed that by this time the trial, its community and patterns of intervention were sufficiently established that the contentious issue of treatment costs and the loss of industry support, did not impact upon recruitment at all.

### Completion work

Towards the end of the research process the trialists had to engage once again with financial considerations to ensure that their trials achieved their aims. All of the trials needed to extend their recruitment periods. Recruitment for FOCUS was slower than expected and took place over three and half years rather than the planned three years. It was, however, possible to accommodate the longer recruitment period within the original funds. With a time-only (i.e. no-cost) extension from MRC, FOCUS exceeded its target of 2100 participants by 35 cases.

The other three trials all required funded extensions from MRC or HTA. With regard to the funding extension which was awarded to HPS, an interviewee explained that it was in fact a calculated element of the original funding strategy. The request for an extension was not made in response to difficulties arising in the research process but a deliberate budgeting plan:

*We always knew that we would have to have a funding extension ... We had only applied for a five or six year grant so it was always anticipated that we would need an extension*. (H-39)

The completion work for TOuCAN and ELEVATE required some reorganisation of their design, as well as the input of additional funds and time. Although recruitment was very successful for TOuCAN, it became evident during the recruitment period that the target of 210 adolescents (70 per arm) randomised into the RCT was not going to be met within the original timeframe. TOuCAN included an additional naturalistic arm wherein up to 70 adolescents who declined to have their care randomised, but wished to contribute to the research, could be followed up. The shortfall in both the trial and the naturalistic arm was attributed to the fact that although the clinicians had referred "quite a lot of young people" (T-34), the trial team was notified about significantly fewer cases than they had anticipated. Even with accrual of 90% of the referred cases, the initial calculations needed reassessment.

*We did a revised power calculation part way through ... and we found that we would still just have enough power if we could get to 50 in each [of the] randomised groups. The rate of recruitment was that we went back to the HTA and said "Look we, we've got this far but we're actually significantly off our recruitment rate. We need an extra year of recruitment." And they gave us an extra year*. (T-27)

With new power calculations the trial target was revised to 55 adolescents per arm, and 75 in the naturalistic arm. A funded extension was awarded by HTA. The target was exceeded with 167 participants recruited to the trial, with an additional 46 adolescents in the naturalistic arm. These figures were achieved in three and a half instead of the intended two years.

Further funds were also granted by HTA in response to revised costings for ELEVATE. At the time of the interviews the trial team were still trying to work out their strategy for completing the trial. One interviewee described the way forward as "currently under debate." (E-12) One of the strata of the trial was proving to be particularly problematic and the team were facing a decision, essentially that they had to "decide whether we're going to try to get a further extension or whether we're going to eat into the follow-up period" (E-13). Another interviewee similarly described the choices that they felt they faced.

*We either will fail or we will revise. ... [We could] keep recruiting for long enough that a slower recruiting rate gets us there but we then have a shorter follow-up for some people*. (E-12)

Further funds were requested and awarded and the trial exceeded the intended sample size of 356 participants for one stratum, recruiting 361 participants, and recruited 325 of the planned 356 participants for the second stratum. The choice to reduce the length of the follow-up period for the second stratum enabled the recruitment period for this group to be extended without another extension of the amount or period of the funding.

## Discussion and conclusion

The aim of this analysis was to explore how a group of trial teams negotiated the UK funding system for clinical trials and how financial considerations in their various forms might shape their research. The trialists and collaborators interviewed for this study described difficult and sensitive activities which went beyond simply making a grant application and balancing the books. Their accounts highlight important ways in which the funding system and broader financial matters affected both the development and subsequent conduct of their trials. They suggest that a major challenge for the teams was often to secure the financial support that they needed, and to conduct the trials with the fewest compromises to their vision of the scientific, clinical and ethical elements of their research. Of particular significance in the accounts of those who resource and manage clinical trials was their experience of dealing with a slow moving, multipart, intricate and at times apparently fragile funding system.

Although there appears to be a clear demarcation of the three different types of costs which need to be met (research, NHS support, and treatment), and delineation of which bodies are responsible for each, the teams in fact described quite individual ways of utilising the system. In part these related to how they had chosen to design and structure their trials. A trial designed to include many patients or expensive drugs required industry support rather than relying on NHS Trusts. A trial backed closely onto standard care procedures required fewer funds for research staff, even though these could have been sought as a legitimate research cost. The trialists' use of the funding system appeared to be as much a product of what they felt was appropriate, given their research design and its relation to the clinical environment in which it was placed, as of the structure of the funding system itself.

An important element in this system for three of the trials was the need to deal with the multiple funders, a tricky and often delicate process in its own right. It often involved nurturing and sustaining the interest of one potential funder whilst engaging with the (potentially different) concerns and priorities of another, a difficult juggling act which commenced sometimes years before the trial could get underway. FOCUS, HPS and ELEVATE all experienced major and ultimately significant funding-related delays, arising from their discussions with potential funders. These delays meant that the circumstances in which the trials were eventually conducted were altered, allowing other research to have been developed or to report in the meantime. Their experience suggests that efforts to facilitate the speedy progress of research through the funding system would be of clear benefit to funders and applicants alike.

Of particular significance was the fact that each of the three main funders had the ability to shape the research in quite significant ways. The public sector research funders (MRC and HTA) intervened directly through the review process for applications for funds to ensure that their perceptions of how the research ought to be conducted were taken into account. The NHS Trusts and PCTs could block expensive research in their area, despite the existence of funding agreements such as the Concordat. The funding sector which raised the most discussion in the interviews was, however, undoubtedly the pharmaceutical industry. The extent of the influence, direct and indirect, that industry appears to have over trials which do not in fact originate within their own sphere was striking.

Collaboration with industry can provide important means to run clinical trials [64,65] and was crucial to the development and ongoing progress of HPS, FOCUS and ELEVATE. This form of support allowed the trialists to finance the drugs necessary for their research, to have funds at their disposal to facilitate important collaborations, and even to provide extra staff or equipment when needed. Interviews with clinical staff in FOCUS collaborating centres suggested that lucrative collaborations with industry trials helped to sustain an infrastructure which was vital to their involvement with a range of public sector funded trials.

The relationship with industry is, however, not without problems. This "uneasy alliance" [5] of potentially competing aims has been likened to "dancing with porcupines" [4,66]. Concerns which have been expressed to date largely relate to the potential for conflict of interests [65,67-70]. The trialists interviewed here were very much aware of the differences between their own research agenda which were focused on developments for patient care, and the commercial agenda of the pharmaceutical companies. Research suggests that they are right to be cautious, demonstrating that links with industry can shape the design and implementation and results of clinical trials [71-73]. Trials that are funded in this way are more likely to produce "pro-industry conclusions" [74]. They are also becoming increasingly prevalent with concerns being expressed that control of the clinical research agenda is passing from academia to industry [75]. Those interviewees who had participated in discussions over the design of their trials used terms such as "stuck to our guns", "held our ground" and "stood firm" to convey the challenges involved in their negotiations. This research suggests that there are also other ways in which the relationship with industry might affect the research process. The withdrawal of funds from FOCUS part-way through the recruitment period highlights a degree of vulnerability for trials which are reliant upon this type of funding. FOCUS is not alone in this experience as a number of trials have been forced to close when funds were withdrawn for commercial reasons [76-79]. It also became clear that there could be direct competition between the trials considered here and other industry-led trials, either for the co-operation of collaborators or for access to eligible patients. In the case of ELEVATE the loss of whole groups of patients from GP practices in their geographical area severely hampered their recruitment processes.

TOuCAN stands alone in this study as a trial which is of little interest to industry. Trials which assess interventions with little or no potential for commercial application are heavily reliant on charitable and public sector funding [3]. Hemminki and Kellokumpu-Lehtinen [7] suggest that the same is true of cutting edge research in areas such as gene therapy and cancer vaccines, with their complexities of intellectual property rights and manufacturing difficulties. The particular needs of non-commercial trials have been acknowledged by the European Union [80]. Although such trials have fewer funding opportunities available to them, it may be that TOuCAN benefited from a simple funding process and did not require complex negotiations with industry. It was the only trial of the four considered here which was not perceived by the interviewees to be affected by financial difficulties. The not-inconsiderable treatment costs associated with in-patient and out-patient care which were assessed within this trial were met within existing contractual agreements between service providers and the referring services. The smooth transition from previous care arrangements to research-led procedures, mapping research onto an established practical and financial framework of referrals and delivery of an intervention, gave this trial a clear advantage. Freed from having to consider complicated practical and political issues of treatment costs, and maintaining the support and interest of industry funders, the central trial team were able to work on engaging and motivating the linchpin collaborators in the important clinical and scientific issues at the heart of the trial.

Once the trials in this study had obtained their necessary finances, they were still faced with financial considerations in the course of their research. The trial teams in this study acted in a number of dynamic ways to address unpredictable financial issues as they arose, and to prevent financial difficulties destabilising their trials. The ELEVATE trialists for instance quickly secured the resources necessary to modify their approach to recruitment and enable additional staff to be deployed where needed.

The teams also had to find the strategies or means to bring their trials to completion. They were not at all unusual in obtaining extensions either in time or money or both. This very common phenomenon amongst clinical trials [81] might occur for a number of reasons. Trials may be under-funded in the first place, because it is so difficult to predict the actual level of funding that will be required, or because circumstances and so costs can change between the application for funds and the recruitment period. TOuCAN required a funded extension because there were fewer eligible adolescents available than had been anticipated. It may also be the case that investigators strategically under-bid in order to secure some funding, even if this is unlikely to be sufficient for the entire trial.

The experiences reported here indicate that a range of skills and a degree of agility are required for negotiations with potential funders and collaborators, and for the ongoing financial balancing act that managing a trial with substantial research budgets entails. Those who are responsible for these onerous tasks receive little, if any, training in this area. The development of systems to support and train researchers in trial processes, such as those being provided by the UK Clinical Research Network [6] would seem to be appropriate. It may also be desirable for public sector funding bodies to demonstrate reciprocal skills and flexibility in assessing the financial plans of research applicants, assisting with the integration of their multi-sourced funds, monitoring and supporting the progress of on-going trials.

This qualitative study was based on four trials which were all identified by their funders as exemplars. It is probable that the extension of this research to a larger number of trials including those considered less successful would have provided additional insights into this complex process. The study was carried out in the UK in 2003–04 and the four trials, each with their own timescale, had encountered the funding process at different times in its evolution. Some changes in resourcing arrangements have been made since the interviews were conducted. There have been moves towards Full Economic Costing for research which may help to increase the transparency of funding decisions and processes. The recent implementation of the EU Clinical Trials Directive may mean that trials require even larger amounts of funding, thus making it "all but impossible to carry out researcher-led studies without the financial and logistical backing of the pharmaceutical industry" [9]. Indeed, the problem of inadequate funding for clinical trials was highlighted by the Academy of Medical Sciences [64], which suggested the need for a new funding structure and the development of an effective research infrastructure to aid efficient organisation of clinical research and training. The UK Clinical Research Network [6] was established in 2005 to provide such an infrastructure, and early in 2006, plans for a single, ring-fenced budget to support the health research currently funded by the MRC and HTA programmes were announced for consultation [82].

The specifics of funding arrangements are bound to differ over time and between different countries. The important general lesson from this study is the need for attention to be paid, by funders and trialists, to the complex practical and financial aspects of planning and conducting a trial, whatever the prevailing funding system. Given the likely influence of the level of funding on the degree of success of clinical trials, it is surprising that this element of the research process is virtually invisible in the research literature. A focus on the issues raised in the financial management of research should be one part of the development and continuing assessment of methods which are aimed at facilitating the conduct of sound clinical research in the future.

## Competing interests

CS holds a fellowship partly funded by MRC. DE, MKC, IR, AMG and AMM all hold research grants for multicentre trials from MRC and, or, the HTA Programme. JG, AMM and RCK have received salary support from MRC trial grants in the past.

## Authors' contributions

The idea for STEPS was jointly conceived by the Principal Investigator, MKC with AMG, VAE, DE, JG, CS, IR, DF, AMM and RCK. CS conducted the interviews and analysis for the qualitative study and with DE and JG took primary responsibility for preparation of the manuscript. All authors contributed to the final manuscript. MKC is guarantor for STEPS, and CS is guarantor for the qualitative study.

## References

[B1] Department of Health, Attributing revenue costs of externally-funded non-commercial research in the NHS. http://www.dh.gov.uk/assetRoot/04/12/52/82/04125282.pdf.

[B2] Partnership Agreement between the Health Departments and the MRC. http://www.mrc.ac.uk/Utilities/Documentrecord/index.htm?d=MRC002125.

[B3] Chalmers I, Rounding C, Lock K (2003). Descriptive survey of non-commercial randomised controlled trials in the United Kingdom, 1980–2002. BMJ.

[B4] Schafer A Medine, Morals and money: dancing with porcupines or sleeping besides elephants. http://www.umanitoba.ca/centres/ethics/articles/article2.html.

[B5] Bodenheimer T (2000). Uneasy alliance: clinical investigators and the pharmaceutical industry. N Engl J Med.

[B6] UK Clinical Research Collaboration. http://www.ukcrc.org.

[B7] Hemminki A, Kellokumpu-Lehtinen PL (2006). Harmful impact of EU clinical trials directive. BMJ.

[B8] Mitchell CD (2006). Harmful impact of EU clinical trials directive: ...while paediatric oncology is being scuppered. BMJ.

[B9] Hanning CD, Rentowl P (2006). Harmful impact of EU clinical trials directive: Trial of alerting drug in fibromyalgia has had to be abandoned. BMJ.

[B10] Watson M (2006). Harmful impact of EU clinical trials directive: ...and so has trial of melatonin in cancer related weight loss.... BMJ.

[B11] Emanuel EJ, Schnipper LE, Kamin DY, Levinson J, Lichter AS (2003). The costs of conducting clinical research. J Clin Oncol.

[B12] Aaronson NK, Visser-Pol E, Leenhouts GH, Muller MJ, van der Schot AC, van Dam FS, Keus RB, Koning CC, ten Bokkel Huinink WW, van Dongen JA, Dubbelman R (1996). Telephone-based nursing intervention improves the effectiveness of the informed consent process in cancer clinical trials. J Clin Oncol.

[B13] Dickinson CJ (1994). Clinical research in the NHS today. J R Coll Physicians Lond.

[B14] Smyth JF, Mossman J, Hall R, Hepburn S, Pinkerton R, Richards M, Thatcher N, Box J (1994). Conducting clinical research in the new NHS: the model of cancer. United Kingdom Coordinating Committee on Cancer Research. BMJ.

[B15] Shea S, Bigger JT, Campion J, Fleiss JL, Rolnitzky LM, Schron E, Gorkin L, Handshaw K, Kinney MR, Branyon M (1992). Enrollment in clinical trials: institutional factors affecting enrollment in the cardiac arrhythmia suppression trial (CAST). Control Clin Trials.

[B16] Morse EV, Simon PM, Besch CL, Walker J (1995). Issues of recruitment, retention, and compliance in community-based clinical trials with traditionally underserved populations. Appl Nurs Res.

[B17] Foley JF, Moertel CG (1991). Improving accrual into cancer clinical trials. J Cancer Educ.

[B18] Ross S, Grant A, Counsell C, Gillespie W, Russell I, Prescott R (1999). Barriers to participation in randomised controlled trials: a systematic review. J Clin Epidemiol.

[B19] Campbell M, Snowdon C, Francis D, Elbourne D, McDonald A, Knight R, Entwistle V, Garcia J, Roberts I, Grant A, (The STEPS Group) Recruitment to randomised trials: Strategies for Trial Enrolment and Participation Study (STEPS). HTA Methodology Programme Monograph.

[B20] McDonald AM, Knight RC, Campbell MK, Entwistle VA, Grant AM, Cook JA, Elbourne DR, Francis D, Garcia J, Roberts I, Snowdon C (2006). What influences recruitment to randomised controlled trials? A review of trials funded by two UK funding agencies. Trials.

[B21] Snowdon C, Elbourne D, Garcia J, the STEPS Study Group (2005). Strategies for Trials Enrolment and Participation Study: Case Studies [abstract]. Clinical Trials.

[B22] Francis D, Roberts I, Elbourne D, Campbell MK, the STEPS study group (2005). Clinical trials as businesses: strategies for trials enrolment and participation study [abstract]. Clin Trials.

[B23] Baines CJ (1984). Impediments to recruitment in the Canadian National Breast Screening Study: response and resolution. Control Clin Trials.

[B24] Taylor KM, Margolese RG, Soskolne CL (1984). Physicians' reasons for not entering eligible patients in a randomized clinical trial of surgery for breast cancer. N Engl J Med.

[B25] Klein MC, Kaczorowski J, Robbins JM, Gauthier RJ, Jorgensen SH, Joshi AK (1995). Physician's beliefs and behaviour during a randomized controlled trial of episiotomy: consequences for women in their care. Can Med Assoc J.

[B26] Fairhurst K, Dowrick C (1996). Problems with recruitment in a randomized controlled trial of counselling in general practice: causes and implications. J Health Serv Res Policy.

[B27] Leach MJ (2003). Barriers to conducting randomised controlled trials: lessons learnt from the Horsechestnut & Venous Leg Ulcer Trial (HAVLUT). Contemp Nurse.

[B28] Heart Protection Study Collaborative Group (2004). Effects of cholesterol lowering with simvastatin on stroke and other major vascular events in 20 536 people with cerebrovascular disease or other high-risk conditions. Lancet.

[B29] Heart Protection Study Collaborative Group (2003). MRC/BHF Heart Protection Study of cholesterol-lowering with simvastatin in 5963 individuals with diabetes: a randomised placebo-controlled trial. Lancet.

[B30] Seymour M (2005). Fluorouracil, Oxaliplatin and CPT-11 (irinotecan), use and sequencing (MRC FOCUS): a 2135- patient randomized trial in advanced colorectal cancer (ACRC)[abstract]. Journal of Clinical Oncology.

[B31] A pragmatic single-blind RCT and health economic evaluation of leukotriene receptor antagonists in primary care at steps two and three of the National Asthma Guidelines (ELEVATE). http://www.hta.nhsweb.nhs.uk/project/1204.asp.

[B32] Gowers SG, Clark A, Roberts C, Shore A, Edwards V, Bryan, Smethurst N, Byford S, Barratt B, Harrington RC Two year outcomes of a randomised controlled trial for adolescent anorexia nervosa (the TOuCAN trial).

[B33] May CR, Williams TL, Mair FS, Mort MM, Shaw NT, Gask L (2002). Factors influencing the evaluation of telehealth interventions: preliminary results from a qualitative study of evaluation projects in the UK. J Telemed Telecare.

[B34] May C, Mort M, Williams T, Mair F, Gask L (2003). Health technology assessment in its local contexts: studies of telehealthcare. Soc Sci Med.

[B35] Hamilton Brown A (2004). Integrating research and practice in the CSAT Methamphetamine Treatment Project. J Subst Abuse Treat.

[B36] Benson AB, Pregler JP, Bean JA, Rademaker AW, Eshler B, Anderson K (1991). Oncologists' reluctance to accrue patients onto clinical trials: an Illinois Cancer Centre study. J Clin Oncol.

[B37] Taylor KM, Feldstein ML, Skeel RT, Pandya KJ, Ng P, Carbone PP (1994). Fundamental dilemmas of the randomized clinical trial process: results of a survey of the 1737 Eastern Cooperative Oncology Group investigators. J Clin Oncol.

[B38] Langley C, Gray S, Selley S, Bowie C, Price C (2000). Clinicians' attitudes to recruitment to randomised trials in cancer care: a qualitative study. J Health Serv Res Policy.

[B39] Grunfeld E, Zitzelsberger L, Coristine M, Aspelund F (2002). Barriers and facilitators to enrollment in cancer clinical trials: qualitative study of the perspectives of clinical research associates. Cancer.

[B40] Fallowfield L, Ratcliffe D, Souhami R (1997). Clinicians' attitudes to clinical trials of cancer therapy. Eur J Cancer.

[B41] Siminoff LA, Zhang A, Colabianchi N, Sturm CM, Shen Q (2000). Factors that predict the referral of breast cancer patients onto clinical trials by their surgeons and medical oncologists. J Clin Oncol.

[B42] Kornblith AB, Kemeny M, Peterson BL, Wheeler J, Crawford J, Bartlett N, Fleming G, Graziano S, Muss H, Cohen HJ, Cancer and Leukemia Group B (2002). Survey of oncologists' perceptions of barriers to accrual of older patients with breast carcinoma to clinical trials. Cancer.

[B43] Joffe S, Weeks JC (2002). Views of American oncologists about the purposes of clinical trials. J Natl Cancer Inst.

[B44] Ross C, Cornbleet M (2003). Attitudes of patients and staff to research in a specialist palliative care unit. Palliat Med.

[B45] Peto V, Coulter A, Bond A (1993). Factors affecting general practitioners' recruitment of patients into a prospective study. Fam Pract.

[B46] Ellis PM, Hobbs MK, Rikard-Bell GC, Ward JE (1999). General practitioners' attitudes to randomised clinical trials for women with breast cancer. Med J Aust.

[B47] Pearl A, Wright S, Gamble G, Doughty R, Sharpe N (2003). Randomised trials in general practice-a New Zealand experience in recruitment. N Z Med J.

[B48] Prout H, Butler C, Kinnersley P, Robling M, Hood K, Tudor-Jones R (2003). A qualitative evaluation of implementing a randomized controlled trial in general practice. Fam Pract.

[B49] de Vaus DA (1991). Surveys in Social Research.

[B50] Wilson K, Roe B, Wright L (1998). Telephone or face-to-face interviews? A decision made on the basis of a pilot study. International Journal of Nursing Studies.

[B51] Smith E (2005). Telephone interviews in healthcare research: a summary of the evidence. Nurse Researcher.

[B52] Breen A, Austin H, Campion-Smith C, Carr E, Mann E (2007). "You feel so hopeless": A qualitative study of GP management of acute back pain. Eur J Pain.

[B53] Rose PW, Ziebland S, Harnden A, Mayon-White R, Mant D, Oxford Childhood Infection Study group (OXCIS) (2006). Why do general practitioners prescribe antibiotics for acute infective conjunctivitis in children? Qualitative interviews with GPs and a questionnaire survey of parents and teachers. Fam Prac.

[B54] Worth A, Boyd K, Kendall M, Heaney D, Macleod U, Cormie P, Hockley J, Murray S (2006). Out-of-hours palliative care: a qualitative study of cancer patients, carers and professionals. Br J Gen Pract.

[B55] Waterman H, Leatherbarrow B, Slater R, Waterman C (1999). Post-operative pain, nausea and vomiting: qualitative perspectives from telephone interviews. J Adv Nurs.

[B56] McCormick KM, McClement S, Naimark BJ (2005). A qualitative analysis of the experience of uncertainty while awaiting coronary artery bypass surgery. Can J Cardiovasc Nurs.

[B57] Ziebland S, Graham A, McPherson A (1998). Concerns and cautions about prescribing and deregulating emergency contraception: a qualitative study of GPs using telephone interviews. Fam Pract.

[B58] Murchie P, Campbell NC, Ritchie LD, Thain J (2005). Running nurse-led secondary prevention clinics for coronary heart disease in primary care: qualitative study of health professionals' perspectives. Br J Gen Pract.

[B59] Sweet L (2002). Telephone interviewing: is it compatible with interpretive phenomenological research?. Contemp Nurse.

[B60] Layder D (1998). Sociological Practice: linking theory and social research.

[B61] Gowers SG (2006). Evidence Based Research in CBT with Adolescent Eating Disorders. Child and Adolescent Mental Health.

[B62] National Institute for Clinical Excellence (2002). Guidance on the use of irinotecan, oxaliplatin and raltitrexed for the treatment of advanced colorectal cancer.

[B63] Dr David Cunningham, Consultant medical oncologist and 27 other cancer care specialists, Colon Cancer Concern NICE decision will reduce life expectancy. The Telegraph.

[B64] Bell J (2003). Resuscitating clinical research in the United Kingdom. BMJ.

[B65] Weatherall D (2003). Problems for biomedical research at the academia-industrial interface. Sci Eng Ethics.

[B66] Lewis S, Baird P, Evans RG, Ghali WA, Wright CJ, Gibson E, Baylis F (2001). Dancing with the porcupine: rules for governing the university – industry relationship. CMAJ.

[B67] Weatherall D (2000). Academia and industry: increasingly uneasy bedfellows. Lancet.

[B68] Montaner JS, O'Shaughnessy MV, Schechter MT (2001). Industry-sponsored clinical research: a double-edged sword. Lancet.

[B69] Moses H, Martin JB (2001). Academic relationships with industry: a new model for biomedical research. JAMA.

[B70] Chopra SS (2003). Industry funding of clinical trials: benefit or bias?. JAMA.

[B71] Djulbegovic B, Lacevic M, Cantor A, Fields KK, Bennett CL, Adams JR, Kuderer NM, Lyman GH (2000). The uncertainty principle and industry-sponsored research. Lancet.

[B72] Fries JF, Krishnan E (2004). Equipoise, design bias, and randomised controlled trials: the elusive ethics of new drug development. Arthritis Res Ther.

[B73] Lexchin J, Bero LA, Djulbegovic B, Clark O (2003). Pharmaceutical industry sponsorship and research outcome and quality: systematic review. BMJ.

[B74] Bekelman JE, Li Y, Gross CP (2003). Scope and impact of financial conflicts of interest in biomedical research: a systematic review. JAMA.

[B75] Patsopoulos NA, Ioannidis JP, Analatos AA (2006). Origin and funding of the most frequently cited papers in medicine: database analysis. BMJ.

[B76] Black HR, Elliott WJ, Grandits G, Grambsch P, Lucente T, White WB, Neaton JD, Grimm RH, Hansson L (2003). Principal results of the Controlled Onset Verapamil Investigation of Cardiovascular End Points (CONVINCE) trial. JAMA.

[B77] Drueke TB, Descamps-Latscha B, Locatelli F (2003). Stopping a medical research project for financial reasons. Nephrol Dial Transplant.

[B78] Psaty BM, Rennie D (2003). Stopping medical research to save money. J Am Med Assoc.

[B79] Lexchin JR (2005). Implications of pharmaceutical industry funding on clinical research. Ann Pharmacother.

[B80] Mayor S (2004). Draft guidance on clinical trials recognises needs of non-commercial research. BMJ.

[B81] Gates S, Brocklehurst P, Campbell MK, Elbourne DR (2004). Recruitment to multicentre trials. BJOG.

[B82] (2006). A Review of UK Health Research Funding. Sir David Cooksey. http://www.hm-treasury.gov.uk/media/56F/62/pbr06_cooksey_final_report_636.pdf.

